# Mood-Reactive Self-Esteem and Depression Vulnerability: Person-Specific Symptom Dynamics via Smart Phone Assessment

**DOI:** 10.1371/journal.pone.0129774

**Published:** 2015-07-01

**Authors:** Peter C. Clasen, Aaron J. Fisher, Christopher G. Beevers

**Affiliations:** 1 Department of Psychiatry and Behavioral Sciences, Stanford University School of Medicine, Palo Alto, CA, United States of America; 2 Department of Psychology, University of California Berkeley, Berkeley, CA, United States of America; 3 Department of Psychology, The University of Texas at Austin, Austin, TX, United States of America; 4 Institute for Mental Health Research, The University of Texas at Austin, Austin, TX, United States of America; West China Hospital of Sichuan University, CHINA

## Abstract

Cognitive theories of depression suggest that mood-reactive self-esteem, a pattern of cognitive reactivity where low self-esteem is temporally dependent on levels of sadness, represents vulnerability for depression. Few studies have directly tested this hypothesis, particularly using intensive data collection methods (i.e., experience sampling) required to capture the temporal dynamics of sadness and self-esteem as they unfold naturally, over time. In this study we used participants’ smartphones to collect multiple daily ratings of sadness and self-esteem over three weeks, in the real world. We then applied dynamic factor modeling to explore theoretically driven hypotheses about the temporal dependency of self-esteem on sadness (i.e., mood-reactive self-esteem) and its relationship to indices of depression vulnerability both contemporaneously (e.g., rumination, sad mood persistence) and prospectively (e.g., future symptomatology). In sum, individuals who demonstrated mood-reactive self-esteem reported higher levels of rumination at baseline, more persistent sad mood over three weeks, and increased depression symptoms at the end of three weeks above and beyond a trait-like index of self-esteem. The integration of smartphone assessment and person-specific analytics employed in this study offers an exiting new avenue to advance the study and treatment of depression.

## Introduction

Depression is characterized by a constellation of symptoms, including persistent sad mood and low self-esteem (e.g., worthlessness), that fluctuate within individuals across time [1,2]. Relatively little is known about the dynamic relationship between these symptoms within the same individual over time. In this study, we explored the dynamic relationship between sadness and self-esteem using multiple daily momentary assessments on participants’ smartphones. We were particularly interested in understanding whether specific temporal dynamics between sadness and self-esteem confer vulnerability to depression.

The idea that emotions, including the emotional symptoms of depression, fluctuate over time is not novel. Contemporary theories of emotion consistently highlight the transient nature of emotional states [3–5]. Moods have been defined as more persistent than emotions [6], but they likewise fluctuate over time (e.g., [7]). Depression has long been characterized as a mood disorder, described by the persistence of a constellation of symptoms, including sadness, anhedonia, and low self-esteem. Despite the fact that a diagnosis requires that these symptoms persist for a period of a least two weeks or longer, there can be substantial fluctuations in symptoms within the same individual over time (e.g., [2]).

Compelling recent evidence using experience sampling methodology (ESM), or daily surveys participants complete in naturalistic settings, suggests that patterns in the way individuals experience emotions over time represent important characteristics that predict emotional well-being, including depression. Kuppens and colleagues (e.g., [8, 9]) for example, have highlighted the role of emotional inertia, technically the first order autocorrelation of emotion ratings (e.g., sadness at (*t*-1) predicting sadness at (*t*)), which the authors interpret as resistance to change in emotional experience (This metric can also be conceptualized as representing greater persistence of an emotion over time.). Specifically, greater emotional inertia in both positive (e.g., happy) and negative (e.g., sad) emotions is associated with greater levels of depression and lower levels of self-esteem and has been shown to prospectively predict depression in adolescence. Furthermore, elevated autocorrelation, variance, and correlation between sets of emotions (e.g., sad, anxious, cheerful, and content) has also been shown to predict the onset of and recovery from depression episodes [10]. This important work is beginning to elucidate temporal features of emotional experience associated with depression vulnerability, particularly in the experience of specific emotions or groups of emotions.

Self-esteem, or an individual’s appraisal of their self-worth [11], similarly varies over time and these fluctuations have been associated with aspects of well being, including depression vulnerability [12–15]. Indeed, temporal dynamics in self-esteem (e.g., variability over time) have previously been shown to predict subsequent depression above and beyond the influence of global trait-like indices of self-esteem (e.g., [15]). These findings suggest that the dynamics of self-esteem, not just global levels, play a role in depression vulnerability.

What remains unclear is how fluctuations in sadness and self-esteem influence each other over time and whether these temporal dynamics are associated with depression risk. For instance, do changes in the moment-to-moment experience of sadness influence subsequent changes to thoughts and feelings about the self or vice versa? Are individuals with temporally dependent sadness and self-esteem more vulnerable for depression? Theoretical models of depression speculate about the temporal predominance of these symptoms. For example, cognitive models of depression posit that individuals who are vulnerable to depression are those for whom sad mood states trigger a pattern of cognitive reactivity, which includes persistent, global, and negative attributions or attitudes about the self (e.g.,[16–19]). According to these models, schematic negative self-referential thinking is the core mechanism underlying the etiology and maintenance of depression. Thus, sad mood represents a predominant symptom that predicts the stability of other key symptoms (e.g., self-esteem) over time and, therefore, the onset, maintenance, or relapse of depression (e.g., [20,21]).

Thus, cognitive theories offer strong predictions about the nature of symptom dynamics in depression. According to the cognitive reactivity hypothesis [18], sadness triggers negative self-referential thinking, including negative self-evaluation. Therefore, we would expect that depression vulnerable individuals are those for whom fluctuations in self-esteem are temporally dependent on their experience of sadness in time. In other words, they are individuals for whom momentary sadness is a good predictor of future low self—esteem. Individuals whose sense of self does not fluctuate with the rise and fall of sad mood should be relatively less vulnerable to depression.

To test this hypothesis, we sampled momentary sadness and self-esteem, multiple times each day for a period of three weeks on participants’ smartphones (This timeframe was selected to reflect the time period assessed by clinicians when determining an MDD diagnosis (i.e., symptoms must be present for two weeks), while providing sufficient data for the planned analyses. Collecting this data on participants’ smartphones represents a feasible and cost effective approach for patients, researchers, and clinicians.). We applied dynamic factor modeling to the resulting multivariate time-series and an automated algorithm to classify individuals based on whether they demonstrated a significant temporal relationship between momentary sadness and future self-esteem, or not. We refer to this relationship as *mood-reactive self-esteem*. At baseline, prior to the smartphone assessment, participants completed a series of measures of constructs previously shown to be associated with depression vulnerability, including global self-esteem, depression symptom severity, and rumination, a theoretically relevant construct reflecting a reactive style of perseverative negative self-referent thinking that is associated with depression vulnerability and low self-esteem [22,23]. At the end of the three-week smartphone assessment, participants provided another measure of depression symptom severity.

Using these data we sought to 1) employ a person-specific, data driven analytic technique to identify individuals who show a pattern of mood-reactive self-esteem; 2) establish convergent validity for this construct by testing whether a) an established and theoretically-relevant risk factor for depression (rumination) predicted membership in this group, and b) mood-reactive self-esteem was related to another dynamic construct associated with depression vulnerability (sad emotional inertia, or autoregressive stability of sad mood); and 3) establish predictive and incremental validity by examining whether mood-reactive self-esteem predicts changes in future depression symptoms above and beyond a baseline estimate of global self-esteem.

We made several predictions in line with cognitive theories of depression: *Hypothesis 1*: A subset of the sample would be classified as expressing mood-reactive self-esteem using person-specific analyses of the ESM data; *Hypothesis 2a*: Higher baseline rumination would predict membership in the mood-reactive self-esteem group, controlling for baseline depression; *Hypothesis 2b*: Mood-reactive self-esteem group membership would be associated with more persistent sadness over three weeks as indexed by autoregressive stability (i.e., sad inertia) of their ESM sadness ratings; and *Hypothesis 3*: Individuals with mood-reactive self-esteem would have increased depression symptoms at the end of three weeks, relative to baseline levels and individuals who do not demonstrate mood-reactive self-esteem. Importantly, we also predicted that mood-reactive self-esteem represents a dynamic process that is not captured by static measures of self-esteem and predicts unique variance in future depression, above and beyond global self-esteem.

Because we were interested in understanding whether mood-reactive self-esteem represents vulnerability for depression, we explored these hypotheses in a community sample that varied along the dimension of depression severity. This design allowed us to characterize mood-reactive self-esteem across this range of functioning, including individuals who are vulnerable for Major Depressive Disorder (MDD) but would be excluded from a design using diagnostic inclusion criteria (e.g., those with sub-threshold symptoms [24]). This sampling approach is also consistent with efforts to articulate mechanisms associated with clinical outcomes from a dimensional perspective [25].

## Materials and Methods

### Sample

Participants were 81 adults recruited from the community in Austin, Texas (see Table 1 for demographic information). The mean age of participants was 28.72 (*SD* = 8.15) and they ranged from 19 to 55 years old. The sample was 59% female. Participants were 53% Caucasian, 22% multiple races, 11% Asian, 7% African American, and 6% did not endorse a race. Across these categories, 35% of the sample was Hispanic. Study inclusion criteria required that participants own a smart phone that could receive text messages and had an Internet browser. There were no specific exclusion criteria. Ninety-three individuals enrolled in the study. Three did not complete the first laboratory visit (due to missed appointment or equipment failure) and, therefore, did not complete the ecological momentary assessment portion. In addition, nine more participants were excluded from analysis due to errors in data collection or they provided insufficient data (see results section for additional information about response rates). The resulting sample included 81 participants. There were no significant demographic differences between those who were included and excluded from analyses.

**Table 1:** Demographics.

		Mean (*SD*)	Range
Age (years)		28.72 (*8*.*15*)	19–55
		Count	Percent
Gender	Male	34	41.98%
	Female	47	58.02%
Race	Asian	9	11.11%
	African American	6	7.41%
	White	43	53.09%
	Multiple	18	22.22%
	None	5	6.17%
Hispanic	Yes	28	34.57%
	No	53	65.43%
		Mean (*SD*)	Range
Depression (CESD^a^)	Baseline	14.59 (*10*.*78*)	0–50
	End of Week 3	13.10 (*11*.*50*)	0–52
Rumination (RRS^b^)	Baseline	12.77 (*6*.*06*)	0–26
Self-esteem (RSES^c^)	Baseline	20.84 (*6*.*09*)	1–30
		Count	Percent
Psychotropic	Yes	71	87.65%
Medication Use	No	10	12.35%

Psychotropic Medications include: Adderall alone (N = 1), Adderall, Mirtazapine & Olanzapine (N = 1), Clonazepam & Citalopram (N = 1), Paxil (N = 1), Prozac (N = 1), Xanax (N = 1), Zoloft (N = 3), and unknown (N = 1).

^a^ Center for Epidemiologic Studies Depression Scale.

^b^ Ruminative Response Scale.

^c^ Rosenberg Self-esteem scale.

Among this sample, the mean depression score (CESD) was 14.59 (*SD* = 10.78, range = 0–50). Using widely accepted clinical cutoff scores on the CESD (i.e., > = 16 [26]), twenty-six participants (32%) reported symptoms consistent with clinical depression at baseline. The mean rumination score (RRS) was 12.77 (*SD* = 6.06, range = 0–26). The mean baseline self-esteem score (RSES) was 20.84 (*SD* = 6.09, range = 1–30). Ten participants (12.35% of sample) reported current use of a psychotropic medication. These included agents for attentional problems (e.g., Adderall), anxiety (e.g., Xanax), and depression (e.g., Paxil, Zoloft) (See Table 1). There was no significant effect of medication use in any of the analyses reported below. There were no significant differences in baseline depression, rumination, self-esteem, or medication usage between those who were included and excluded from analyses based on availability of ESM data (see above).

### Ethics Statement

The Institutional Review Board at the University of Texas at Austin approved all study procedures and materials and all participants provided signed informed consent.

### Materials

#### Demographic Survey

Participants completed a demographic questionnaire that included questions about age, gender, race/ethnicity and medication use.

#### Rosenberg Self-Esteem Scale (RSES)

The RSES [27] is a commonly used, 10-item measure of global self-esteem. This measure has well-established reliability and validity in general adult samples [28].

#### Center for Epidemiologic Studies Depression Scale (CESD)

The CESD [29] is 20-item measure of depression symptoms developed for use in the general population. This measure has well-established reliability and validity in general adult samples [29].

#### Ruminative Response Scale (RRS)

The RRS [30] is a 10-item scale that measures an individual’s tendency to ruminate, or a perseverative style of self-referent thinking about negative emotions. The RRS provides a measure of rumination that is not confounded with depression symptoms. Previous reports indicate good internal reliability and predictive validity [30].

#### Experience sampling method (ESM)

ESM was conducted using fully automated proprietary web-based software administered on participants’ smartphones. This software was designed by one of the authors (PCC), developed in partnership with a commercial web-developer, and extensively tested prior to implementation. Because the software was web-based, participants were not required to download software onto their phones, the service was available across smartphone platforms (e.g., iPhone OS, Android), and data was stored securely on a remote server.

Participants were asked to complete 105 surveys over a three-week period (five per day) on their phones. Surveys were sent at pseudo-random intervals during hours participants specified as their normal waking hours, with the restriction that no two alerts could arrive in the same hour. Text alerts provided links to each survey, which was administered through a secure session on their phone’s web-browser. An initial page encouraged participants to complete the survey immediately, except if they were driving or doing any other activity where it was unsafe to interact with their phone. They then completed a series of three randomly ordered questions (each presented individually, one at a time):

1. How sad do you feel right now?
2. How do you feel about yourself right now?
3. How aroused (or activated) do you feel right now?

**Fig 1:**
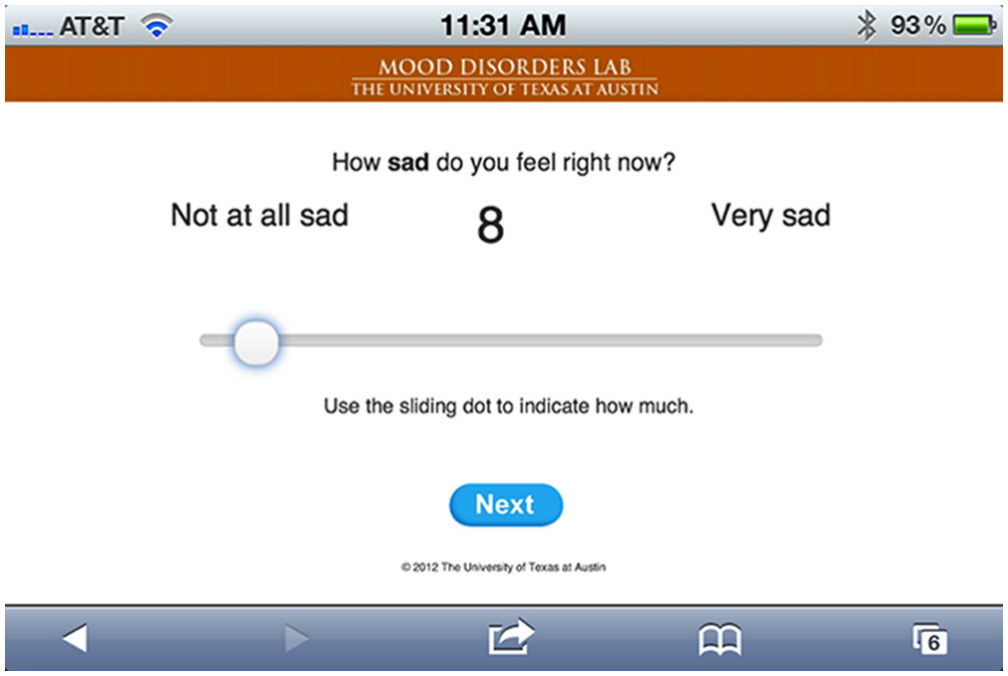
Experience sampling method survey (sad mood item) delivered via smartphone.

These first two questions map onto the symptoms of interest: sad mood and self-esteem (Single item assessments of self-esteem have previously demonstrated adequate reliability and validity [28]. In the current study, the mean of repeated assessments of self-esteem using a single item was strongly correlated with a trait-like index of self-esteem (RSES) sampled at baseline (*r* = 0.58, *p* < 0.0001)). The third question was included to control for differences in momentary levels of arousal during the planned analyses (Arousal represents an important feature of emotional experience [31]. Low arousal is also an important symptom of depression [32]. Because we were interested in the relationship between sadness and self-esteem independent of experiential- or depression-related differences in arousal, we chose to sample this variable so we could control for it in the planned analyses.).Responses were provided using a visual analog slider scale (see Fig 1. Anchor points were provided for each question (e.g., “not at all sad”, “very sad”). If participants missed 5 surveys in a row, the received an automated email encouraging them to respond to all surveys and contact information to reach research staff for questions or concerns.

### Procedure

Participants were recruited via Internet advertisements (e.g., Craigslist). After responding to the advertisement they corresponded with research staff to confirm inclusion criteria. Eligible participants were invited to the Mood Disorders Laboratory at The University of Texas at Austin to complete the baseline assessment. During this assessment they completed the self-report measures (RSES, CESD, RRS) via secure Internet-based data collection software (REDCap;[33]) as well as a series of assessments that are not relevant to the current report.

Next, they enrolled in the ESM site, received a practice text message, and had the chance to practice completing a survey in the presence of trained research staff. This allowed trained research staff to provide additional instructions about the items (e.g., clarifying the specific meaning of arousal (activation) in this context) and troubleshoot any technical problems. At the end of this appointment they were informed that they would receive ESM surveys daily, five-times per day, starting the next day. Research staff explained that they would also receive an invitation to complete the CESD via the REDCap online software at the end of the three-week ESM.

All participants were paid $30 for the baseline assessment. They were told that they would receive additional compensation after the three-week ESM survey period based on the percentage of surveys completed ($15 if they completed more than 75%, $10 if they completed more than 50%, and $5 if they completed less than 25%). They were also told that participants who completed more than 90% of surveys would be enrolled in a raffle to receive a $10 gift card to an online retailer (amazon.com).

Finally, participants had a chance to ask any questions before leaving the baseline assessment. Research staff monitored participant response rates during the ESM survey period and contacted those participants who fell below a 50% response rate to offer assistance and encourage participation. Otherwise, contact was minimized throughout the ESM period. When participants completed the three-week ESM period they were invited back to the lab to be compensated according the performance schedule outlined above.

### Approach to statistical modeling

In order to examine the dynamic relationships between sadness, and self-esteem, while controlling for arousal, at the individual level, we employed dynamic factor modeling [34]—a vector autoregressive (VAR) methodology that utilizes a structural equation modeling framework for assessing contemporaneous correlations and time-lagged regressions in multivariate time series. While these analyses can be carried out in widely available statistical packages, a time-lagged covariance matrix, known as a block-Toeplitz matrix, is required as the input in order to properly model temporal covariances in individual time series. Consistent with other multivariate general linear model procedures—such as multiple regression—correlations and regression parameters are estimated simultaneously and statistically control for the presence of each other in the model (cf. [35]).

Dynamic factor modeling assumes a roughly even spacing between observations in time, so that regression coefficients are not artificially weakened or strengthened by varyingly longer or shorter temporal spacing. The present approach handled uneven spacing by partialing out the variance attributable to temporal distance between observations. A mean-centered vector of time-lag values (in minutes) was affixed to each individual’s data, in addition to the interactions between lag-time and arousal, sadness, and self-esteem (also mean-centered). This approach allowed us to directly model the effect of lag-time variability on coefficients in the model. Because these data were mean-centered within each individual, the presence of the effects of lag-time were effectively partialed out, allowing us to interpret the autoregressive and cross-lagged main effects of arousal, sadness, and self-esteem as the effects these variables exert when lag-time is held at its mean value.

For each individual a 7 x 105 array—consisting of the consecutive observations for arousal, sadness, and self-esteem across the study period—were used to estimate block-Toeplitz matrices. Each resulting covariance matrix contained the contemporaneous and time-lagged covariance between variables at time (*t*) and times (*t*-*n*), where n is a designated number of lags. The present study employed a VAR (1) model, which estimated a single lag. Thus, for each participant we generated a model containing a set of contemporaneous correlations between arousal, sadness, and self-esteem at times (*t*) and (*t*-1), respectively, and regressions of all variables at time (*t*) on time (*t*-1)—in addition to the centered time-lag variable and time-lag x study variable interactions.

**Fig 2:**
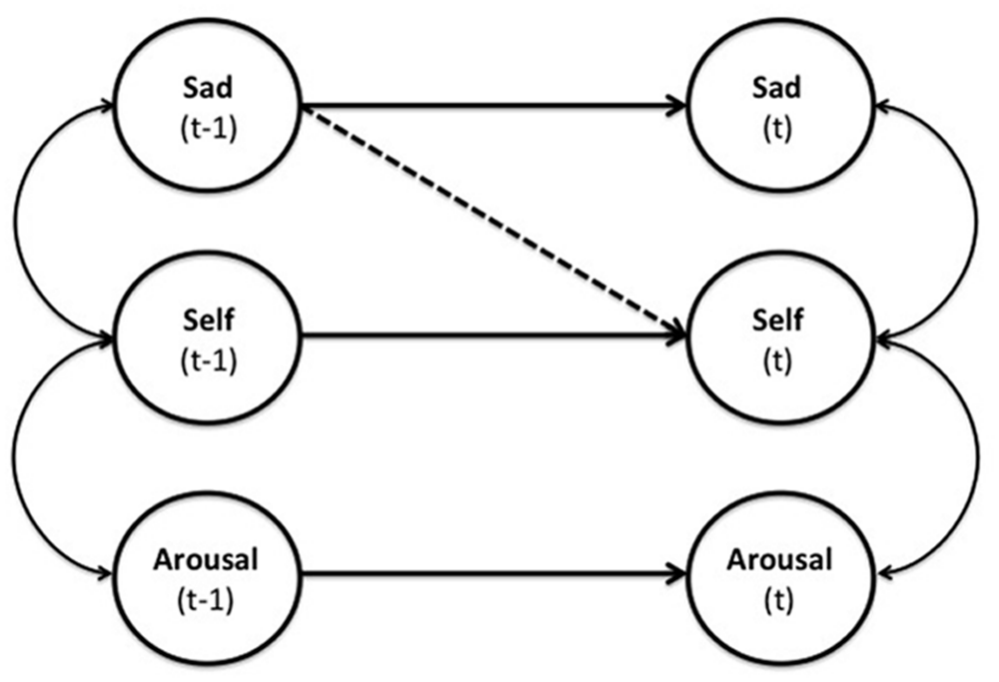
Conceptual model for the dynamic relationships between arousal, sadness, and self-esteem. Straight lines are time-lagged regression parameters and curved lines are contemporaneous correlations. The dashed line indicates the temporally dependent regression of self-esteem on sadness (i.e., mood-reactive self-esteem).

Once covariances were estimated, they were inputted into LISREL (v8.80)[36]. A model with a set of initial conditions was established that included all autoregressions and contemporaneous correlations. In order to explore the idiosyncratic structure of the individual dynamic models, an automatic search function utilizing Lagrange multiplier tests [37] was employed to identify cross-lagged regression parameters for each of the 81 participants. Thus, each individual’s dynamic factor model was allowed to have an idiosyncratic set of cross-lagged regressions. Once each model was constructed, standardized coefficients were extracted from each individual model for use in regression analyses reported below; the absence of a given cross-lagged regression was indicated by a missing value for these analyses. Fig 2 presents the conceptual model for the dynamic relationships between arousal, sadness, and self-esteem.

## Results

### ESM Response Rates & Feasibility Data

Overall participants were highly compliant with the ESM procedure. Eighty-seven participants successfully enrolled in the ESM during the baseline assessment. Four individuals used smartphone operating systems that were not compliant with the ESM software (e.g., customized Google internet phones). Two individuals did not respond to any surveys. Of the 81 participants who did respond, they completed an average of 77.68 (*SD* = 19.75) or 74% of surveys. The distribution of response rates across the sample was negatively skewed; median number of surveys completed was 80, 75% of the sample completed 71 or more surveys, and 90% of the sample completed 52 or more surveys (roughly half of the surveys sent). Response rates did not vary significantly by demographic factors (i.e., no differences as a function of age, gender, race, or ethnicity). Moreover, response rate was not related to severity of depression (CESD): *r* = -.09, p = 0.43; level of rumination (RRS): *r* = -.20, *p* = 0.08; or current use of psychotropic medications: *r* = 0.02, *p* = 0.87.

### Determining the target population: Mood-reactive self-esteem

To determine whether a sub-population of the sample exhibited mood reactive self-esteem (*Hypothesis 1*) we identified individuals who exhibited a cross-lagged parameter of self-esteem (*t*) on sadness (*t*-1). Twenty-three of the 81 participants (28%) exhibited this relationship and were designated as the target group. (As noted above, LISREL was programmed to perform an automatic search using the Lagrange multiplier test. This function tests the relative change in model chi-square that accompanies the addition of a given parameter. LISREL was programmed to disallow any regression parameters that failed to significantly improve model fit and include those parameters that improved fit (on an iterative basis, starting with the strongest parameter). Thus, for the 23 participants exhibiting mood-reactive self-esteem, the addition of a temporally-lagged regression of self-esteem (*t*) on sadness (*t*-1) provided a significant improvement to model fit.).

### Determining the comparison group: Self-esteem reactive sadness

In order to demonstrate the specificity of the directional relationship between sadness and self-esteem, we also identified individuals who exhibited the inverse cross-lagged relationship between sadness (*t*) on self-esteem (*t*-1) (i.e., Self-reactive sadness). Twenty-eight of the 81 participants (35%) exhibited temporally dependent regression parameters of sadness on self-esteem. Of note, only six participants exhibited both sadness on self-esteem *and* self-esteem on sadness cross-lagged relationships.

### Mood-reactive self-esteem and baseline ruminative style

Next, we examined whether rumination predicted membership in the mood-reactive self-esteem group, controlling for baseline depression symptoms (*Hypothesis 2a*), using a hierarchical generalized linear regression (results are reported in Table 2). Baseline depression was entered first as a predictor of target group membership in a logistic regression, rumination and baseline self-esteem were added in the second and third steps. Depression was initially a significant predictor of group membership; however, after adding rumination to the model depression was no longer a significant predictor, while rumination was a significant predictor of target group membership. Baseline self-esteem was not a significant predictor of group membership and its addition to the model did not affect the non-significant effect of depression or the significant effect of rumination. Thus, it appears that rumination specifically (and not depression or self-esteem more broadly) is a significant predictor of mood-reactive self-esteem.

**Table 2:** Odds of mood-reactive self-esteem group membership, predicted by baseline depression, ruminative style, and baseline self-esteem.

	OR	SE	*z* value	*p* value	deviance	Δ deviance
Step 1:						
Depression	1.70	1.28	2.15	.03	91.12	
Step 2:						
Depression	1.25	1.32	2.15	.03		
Rumination	2.30	1.38	2.59	.009	83.13	7.99
Step 3:						
Depression	1.66	1.51	1.23	.22		
Rumination	2.57	1.40	2.80	.005		
Baseline Self-esteem	1.72	1.53	1.27	.20	80.01	3.12

In order to test the specificity of these findings to the mood-reactive self-esteem group, we reran this model to predict membership in self-esteem reactive sadness group. None of these variables predicted membership in the comparison group (self-esteem reactive sadness): Depression (*b* = .03, *95% CI* = (-0.05, 0.11), *SE* = .04, *t(76)* = 0.74, *p* = .46), rumination (*b* = .005, *95% CI* = (-0.11, 0.12), *SE* = .06, *t(76)* = 0.083, *p* = .93), and baseline self-esteem (*b* = -.008, *95% CI* = (-0.15, 0.13), *SE* = .07, *t(76)* = 0.11, *p* = .91).

### Relationship to autoregressive stability (i.e., persistence) of sadness

To determine whether mood-reactive self-esteem was associated with autoregressive stability in sadness (*Hypothesis 2b*), we regressed the standardized autoregression coefficients from the individual dynamic factor models on target group membership (results are reported in Table 3). Group was a significant predictor of autoregressive stability, accounting for 22% of the variance in the relationship between sadness (*t*) and sadness (*t*-1). The addition of baseline measures of depression and self-esteem to the model did not impact this effect.

**Table 3:** Degree of autoregressive stability of momentary depression, predicted by baseline depression, mood-reactive self-esteem group status, and baseline self-esteem.

	coefficient	SE	*t* value	*p* value	R^2^	Δ R^2^
Step 1:						
Depression	.0005	.002	.19	.85	.0005	
Step 2:						
Depression	-.002	.002	-.99	.33		
MRSE	.245	.052	4.74	< .001	.23	.2295
Step 3:						
Depression	-.003	.003	-.87	.39		
MRSE^a^	.240	.053	4.55	< .001		
Baseline Self-esteem	.0003	.006	.05	.96	.23	0

^a^ Mood-reactive self-esteem group membership.

Thus, those with mood-reactive self-esteem exhibited significantly more persistent levels of sadness from moment-to-moment, even when controlling for baseline depression and self-esteem. Importantly, the inverse was not true: Self-esteem reactive sadness was not associated with temporal stability in sadness (*b* = .005, *95% CI* = (-0.12, 0.13), *SE* = .07, *t(80)* = 0.079, *p* = .94).

### Mood-reactive self-esteem and change in depression symptoms

Finally, we assessed whether individuals with mood-reactive self-esteem were more likely to experience increased depression symptoms over the three-week study, controlling for baseline levels (*Hypothesis 3*). We created a depression symptom change score by subtracting baseline CESD scores from those measured at the end of the study (i.e. 3 weeks later). Additionally, we opted for a baseline-adjusted analysis, having demonstrated that initial levels of depression were significantly related to membership status in the target group (mood-reactive self-esteem). We therefore regressed change in depression on baseline depression, baseline self-esteem and mood-reactive self-esteem group membership (results are reported in Table 4). Target group membership significantly predicted change in depression, as did baseline levels of depression and self-esteem Thus, those who demonstrated mood-reactive self-esteem had significantly increased depression symptoms over the three-week period, controlling for baseline depression. Mood-reactive self-esteem predicted unique variance in future depression, above and beyond the influence of global self-esteem. Once again, the opposite effect (for self-esteem reactive sadness) was not a significant predictor of change in depression (*b* = .12, *95% CI* = (-5.29, 5.53), *SE* = 2.76, *t(64)* = 0.044, *p* = .96).

**Table 4:** Change in depression (from baseline), predicted by baseline depression, baseline self-esteem and mood reactive self-esteem group status.

	coefficient	SE	*t* value	*p* value	Cohen’s *d*
Baseline Depression	-.65	.14	-4.73	<.001	1.18
Baseline Self-esteem	-.78	.23	-3.39	.001	.85
MRSE^a^	7.02	2.15	3.26	.002	.82

^a^ Mood-reactive self-esteem group membership.

**Fig 3:**
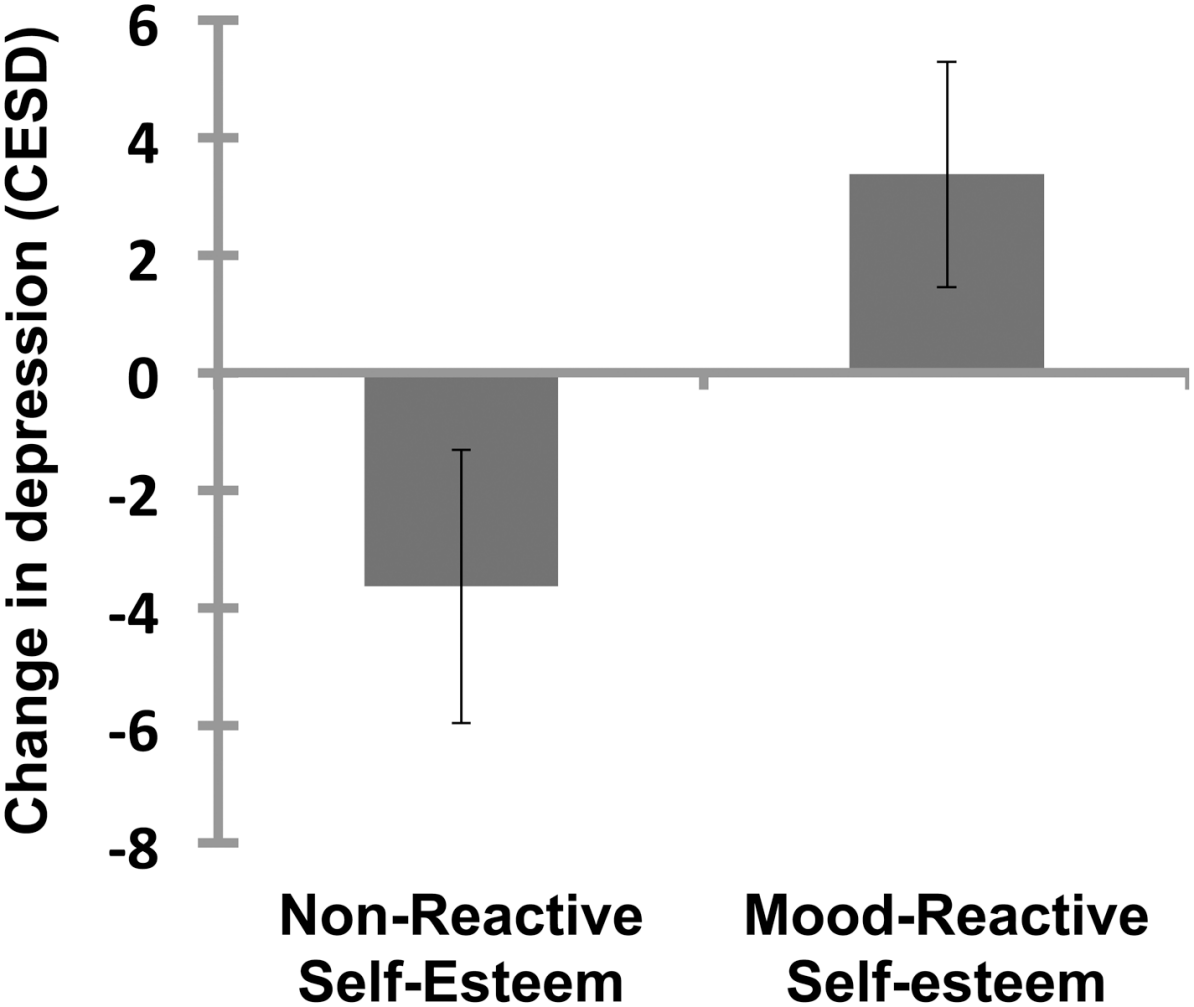
Change in depression over study period between those with and without mood-reactive self-esteem (baseline depression and baseline self-esteem held at mean values). Group difference estimate = 7.015, SE = 2.15, *t(64)* = 3.26, *p* = .002, *d* = 0.82. Error bars reflect 95% confidence intervals.

Fig 3 presents the relative change in depression over the study period in the target and comparison groups. Of note, follow-up data were missing for 10 (12%) of the sample, of which 1 participant was in the target group.

## Discussion

Cognitive theories posit that cognitive reactivity is a key vulnerability for depression (e.g., [19]), but few studies have examined this hypothesis longitudinally with intensive data collection methods necessary to capture the dynamics of cognitive reactivity as it unfolds naturally, over time. Using multiple daily assessments on participants’ smartphones and person-specific dynamic factor modeling, this study sought to elucidate the temporal relationship between sadness and self-esteem and relate these dynamics to indices of depression vulnerability. Results support cognitive theories of depression and indicate that depression vulnerability is associated with mood-reactive self-esteem, or a pattern of cognitive reactivity where momentary sadness predicts future self-esteem. Higher trait rumination predicted which individuals expressed mood-reactive self-esteem, controlling for initial depression. Moreover, individuals with mood-reactive self-esteem demonstrated more persistent sadness over time. Given that these constructs have previously been associated with depression risk, these findings establish convergent validity for the role of mood-reactive self-esteem in depression risk. Most notably, mood reactive self-esteem also prospectively predicted increased depression symptoms above and beyond a trait-like index of global self-esteem, establishing both predictive and incremental validity for this construct.

These findings suggest that among individuals who may be vulnerable for depression, self-esteem is temporally dependent on affective experience. Cognitive theories have long argued that affective episodes, and specifically episodes of sadness, trigger schematic negative thinking about the self in depression (e.g., [16–18]). Findings from this study indicate that sadness is not merely a trigger, but can be a barometer for negative self-referent thinking in depression. In other words, individuals with higher levels of rumination, who experience more persistent sadness over time, and experienced greater increases in depression across time (all indices of depression vulnerability) appear to be more susceptible to the influence of recent affective experience when making momentary self-evaluations, whereas individuals less vulnerable to depression are not.

Our findings differentiate mood-reactive self-esteem from indices of global self-esteem. Global self-esteem is typically measured at a single time point and items ask individuals to aggregate self-evaluative views across time. Low global self-esteem is a risk factor for depression in the literature (e.g., [15,38]) (and in this study); however, it does not appear to be associated with mood-reactive self-esteem: In this sample, baseline global self-esteem did not predict who expressed mood-reactive self-esteem. Moreover, global and mood-reactive self-esteem predicted unique portions of the variance in future depression. Thus, it appears that low global and high mood-reactive self-esteem represent independent vulnerability factors for depression.

Elucidating the mechanisms underlying mood-reactive self-esteem is an important future direction. Cognitive theories suggest that biases in cognitive control during episodes of sad mood (e.g., difficulty disengaging attention from sad information, rumination) represent key mechanisms that could account for reactive self-esteem [16–18]. For example, sad mood may provide fuel for high-trait ruminators to engage in negative self-referent thinking and consequently experience diminished self-esteem [39]. The fact that baseline trait rumination prospectively predicted mood-reactive self-esteem in this study offers preliminary support for this hypothesis.

Mood-state dependent cognitive control biases may also underlie mood-reactive self-esteem. Depression vulnerable individuals demonstrate a host of mood-state dependent cognitive biases, including preferential memory for mood congruent information and dysfunctional attitudes about themselves (see [40] for review). In line with cognitive theories, these biases may activate a network of negative, self-referential schematic processing, in response to sad mood, that ultimately degrades self-esteem. If so, efforts to correct these biases (e.g., cognitive bias modification [41]) may be expected to disrupt mood-reactive self-esteem. It is important to consider that such training may only be successful if it is also mood-state dependent (e.g., occurs in the context of a sad mood provocation).

Interventions aimed at the prevention and treatment of depression can take advantage of the methods implemented in this study. Smartphones are rapidly becoming ubiquitous in the general population (e.g., [42,43]) and there is increasing optimism about leveraging this technology for mental health care (e.g., [44,45]). The analytic framework applied in this study can be fully automated and has the distinct advantage of being person-specific [46]. Thus, these tools are scalable to many different environments where researchers and clinicians are interested in tracking vulnerable individuals, particularly individuals who may not yet have experienced a clinical depression.

These methods are also portable to the treatment setting, and may play an important role in ongoing assessment during treatment for depression. Cognitive therapies, including traditional cognitive therapy and mindfulness-based cognitive therapy, were developed to alter core schematic relationships between sad mood and negative thoughts about the self (e.g., [47,48]). Tracking these specific variables during a therapeutic intervention may provide important feedback for the clinician and patient about changes in underlying processes that precede general symptom remission (e.g., diminished mood-reactive self-esteem). Moreover, these tools may be implemented remotely following therapy to monitor risk for relapse.

Of course, this study had a number of important limitations. First, we limited recruitment to individuals who currently had a smartphone, which may restrict the generalizability of our findings. Recent surveys indicate that 91% of Americans use mobile devices, and between 55–81% of individuals aged 18–55 (the range included in this sample) use smartphones [42]. Nevertheless, future work should consider ways of 1) providing this technology to individuals without smartphones, or 2) adapting ESM procedures to leverage more ubiquitous technologies (e.g., “non-smart” mobile phones; adapting the methods for use with other devices (e.g., tablets); or using email alerts in conjunction with personal computers).

Second, we did not sample other symptoms of depression. Future work should consider ways to capture momentary measures of these symptoms (e.g., anhedonia, sleep disturbance) to examine a broader range of symptom dynamics in depression. This is particularly important given the long acknowledged issue of symptom heterogeneity in Major Depressive Disorder (MDD). Based on DSM-5 diagnostic criteria there are 70 permutations of symptoms that can constitute a diagnosis. Researchers and clinicians speculate that this inherent heterogeneity obscures efforts to identify specific underlying mechanisms and apply appropriate treatments (for review [49]; see also [50]).

A better understanding of the person-specific dynamics between depression symptoms may provide novel insights that help resolve this long-standing issue. For example, for some individuals a lack of sleep may play a predominant role in the downstream experience of sadness and low self-esteem, whereas for others anhedonic behavioral withdrawal predominates. If properly assessed, these person-specific profiles could inform treatment planning (e.g., cognitive behavioral therapy for insomnia and behavioral activation, respectively). These predictions are consistent with emerging network theories of psychopathology that characterize disorders as systems of causal relationships between symptoms (e.g., [51–53]). This framework also easily accommodates other symptoms that fall outside the traditional purview of MDD, but may be clinically and/or epidemiologically relevant (e.g., anxiety, substance use).

Third, we did not design this study to make strong conclusions about the discriminant validity of mood-reactive self-esteem with respect to MDD. Therefore, it is possible that mood-reactive self-esteem represents a more general process associated with various forms of psychopathology. Some may see this as a limitation; we see it as a future direction. The idea that mood-reactive self-esteem may be more broadly associated with psychopathology a) does not detract from the demonstrations of convergent, predictive, and incremental validity with respect to depression vulnerability in this study and b) is consistent with broader efforts to move away from studying processes based on diagnostic category and towards processes that offer increased precision in predicting person-specific outcomes, identifying targets for intervention, and facilitating translational research on biological pathology (e.g., neural mechanisms) [25].

Despite these limitations, we believe this study is an important first step towards understanding symptom dynamics among depression vulnerable individuals. Our findings highlight the role of mood-reactive self-esteem in depression and link this construct to several indices of depression vulnerability, including rumination, the persistence of sad mood, and elevated future depression symptoms. As outlined in this discussion, these findings and the methods implemented in this study offer many exciting opportunities to pursue future theoretical and applied research. The ultimate goal of this work is to provide better insight into the mechanisms that cause and maintain depression so that prevention and treatment efforts can be applied with maximal efficiency and effectiveness.

## Supporting Information

S1 File Dataset. (TXT)
